# Efficacy of Paraffin Wax Bath with and without Joint Mobilization Techniques in Rehabilitation of post-Traumatic stiff hand

**Published:** 2013-04

**Authors:** Fozia Sibtain, Asghar Khan, Syed Shakil-ur-Rehman

**Affiliations:** 1 Dr. Fozia Sibtain, Assistant Professor, Riphah College of Rehabilitation Sciences, Riphah International University, Islamabad, Pakistan.; 2 Dr. Asghar Khan, Associate Professor, Riphah College of Rehabilitation Sciences, Riphah International University, Islamabad, Pakistan.; 3 Dr. Syed Shakil-ur-Rehman, Assistant Professor, Riphah College of Rehabilitation Sciences, Riphah International University, Islamabad, Pakistan.

**Keywords:** Post-traumatic stiff hand, Joint mobilization techniques, Paraffin wax bath

## Abstract

***Objective:*** Post-traumatic stiff hand is common a condition which causes pain and disability, the paraffin wax bath and joint mobilizations have the key role in its rehabilitation. We conducted the present study to determine the efficacy of paraffin wax bath with mobilization techniques compared with joint mobilization alone.

***Methodology: ***This single blind randomized control trial was conducted on 71 patients in department of physical therapy and rehabilitation, Riphah International University Islamabad, and patients with post-traumatic stiff hand after distal upper extremity fractures, were included. The patients were randomized into two groups: the joint mobilization techniques with paraffin wax bath were included in group A, and joint mobilization techniques without paraffin wax bath in group B. The study variables were pain score on visual analogue scale (VAS) 0/10, thumb function score (TFS) and passive range of motion (PROM) of wrist flexion, extension, radial and ulnar deviation, and were compared at baseline and at completion on plan-of-care after six weeks.

***Results:*** Seventy one patients with post-traumatic stiff hand were enrolled and placed randomly into two groups. The baseline characteristics were similar in both groups. Six week after intervention, patients in group A had more improvement in pain score (p=0.001), TFS (p=0.003), and PROM of wrist flexion (p=0.002), extension (p=0.003), radial deviation (p=0.013), and ulnar deviation (p=.004), as compared to group B. However, in group B the improvement was less in pain score (p=0.104), TFS (p=0.520), and PROM of wrist flexion (p=0.193), extension (p=0.1081), radial deviation (p=0.051), and ulnar deviation (p=.168), as compared to group A.

***Conclusion:*** Paraffin wax bath with joint mobilization techniques are more effective than mobilization techniques without paraffin wax bath in the rehabilitation of post traumatic stiff hand.

## INTRODUCTION

The post-traumatic stiff hand commonly occurs in post traumatic upper extremity (UE) fractures, and is clinically complex problem. It generally results in common symptoms of edema, immobility and pain. The post-traumatic stiff hand generally leads to disuse of hand function, due to restricted range of motion and loss of muscle strength. The physical therapists rehabilitate the patients with post-traumatic stiff hand by joint mobilization techniques, stretching and strengthening exercises.^1^ If the patients with post-traumatic stiff hand are not rehabilitated, they will develop contractures in hand muscles and will result in a position of dysfunction. The physical therapy plan of care is based on physical examination, includes evaluation of PROM, muscle strength, edema, gross sensation, bone healing, and adhesions. The common goals of physical therapy management of post-traumatic stiff hand are to manage pain, increase PROM, and muscle strength.^2^

The improvement in joint PROM is the key component of physical therapy management, due to musculotendinous tightness. The joint mobilization techniques are used to improve joint PROM, by producing passive glides with distraction between the articular surfaces of hand joints to manage pain, break adhesions, and improve joint PROM. The grade I and II are used to manage pain and grade-III for improvement in joint PROM.^3^

The paraffin wax bath is commonly used as effective remedy to improve circulation and promotes relaxation.^4^ Both hands and feet are most common segments to be treated with paraffin wax bath in physical therapy. Paraffin Wax bath treatment followed by active hand exercise resulted in significant improvements of range of motion.^5^

## METHODOLOGY

This study is a randomized clinical trial on 71 patients, with the mean age of 39.5 years with minimum age 21 years and maximum 52 years conducted, in department of physical therapy and rehabilitation, Riphah International University Islamabad. This interventional study was conducted from November, 2010 to September, 2012. The inclusion criteria were age range from 20-60 years, pain, loss of PROM, with history of trauma and distal upper extremity fractures. The patient with age less than two years and more than 60 years with any non-traumatic cause of fractures were considered as exclusion criteria. The study variables were measured and documented at the baseline including, age, gender, dominant hand, hand involved, prior level of activity, pain intensity score, Thumb function score, and PROM of wrist flexion, extension, radial and ulnar deviation (Table-I). The PROM was measured by goniometer in sitting position.

Thirty six patients were placed randomly in group A, and treated with joint mobilization techniques and paraffin wax bath, and 35 patients were included in group B and were treated with joint mobilization techniques alone. All the patients of both the groups were treated 4 days per week for 6 weeks. The paraffin wax bath was applied for 20 minutes prior to every physical therapy session and followed by joint mobilization techniques including glides of the articular surfaces in sitting position at 8-12 glides at every joint of the hand and wrist. The joint mobilization grade-I and grade-II were used for pain management and relaxation, while grade-III for improvement in the PROM of hand and wrist. The study variables were also calculated at the completion of six weeks physical therapy treatment program.

Statistical analysis was done by SPSS version-20 and the paired t-test was applied at 95% level of significance to calculate the p values for pain intensity score on VAS 0/10, TFS and PROM of wrist flexion, extension, radial and ulnar deviation (Table-II).

**Table-I:** Baseline characteristics of 71 patients with post-traumatic stiff hand

*Characteristic*	*Group-A, joint mobilization with paraffin wax bath (n=36)*	*Group-B, joint mobilization without paraffin wax bath (n=35)*	*Total*
Male patients	21 (47.72%)	23 (52.27%)	44 (61.97%)
Female patients	15 (55.55%)	12 (44.44%)	27 (38.02%)
Rt hand dominant	35 (51.47%)	33 (48.52%)	68 (95.77%)
Lt hand dominant	01 (33.33%)	02 (66.66%)	03 (04.22%)
Rt hand involvement	16 (57.14%)	12 (42.85%)	28 (39.43%)
Lt hand involvement	24 (55.81%)	19 (44.18%)	43 (60.56%)
Sedentary life style	06 (35.29%)	11 (64.70%)	17 (23.94%)
Active life style	30 (55.55%)	24 (44.44%)	54 (76.05%)

**Table-II:** Clinical and functional changes at six weeks in patients with post-traumatic stiff hand

*Characteristic*	*Group-A, joint mobilization with paraffin wax bath (n=36)*		*Group-B, joint mobilization without paraffin wax bath (n=35)*	
	*Std.deviation*	*p-value*	*Std.deviation*	*p-value*
Pain score	1.135 + 0.359	0.001	0.527 + 0.166	0.104
Thumb function score	1.178 + 0.372	0.003	0.707 + 0.223	0.520
PROM wrist flexion	0.948 + 0.300	0.002	0.674 + 0.213	0.193
PROM wrist extension	0.875 + 0.276	0.003	0.966 + 0.305	0.081
PROM wrist radial deviation	1.337 + 0.422	0.013	0.843 + 0.226	0.051
PROM wrist ulnar deviation	0.816 + 0.258	0.004	0.843 + 0.266	0.168

## RESULTS

Seventy one patients with post-traumatic stiff hand were enrolled and placed randomly into two groups. The baseline characteristics were similar in both groups. Six week after intervention, patients in group A had more improvement in pain score (p=0.001), TFS (p=0.003), and PROM of wrist flexion (p=0.002), extension (p=0.003), radial deviation (p=0.013), and ulnar deviation (p=.004), as compared to group B. However, in group B the improvement was less in pain score (p=0.104), TFS (p=0.520), and PROM of wrist flexion (p=0.193), extension (p=0.1081), radial deviation (p=0.051), and ulnar deviation (p=.168), as compared to group A. Table-II.

## DISCUSSION

In our study, the base line measurements of study variables were matched with measurements after six weeks of physical therapy intervention, including joint mobilization with paraffin wax bath in group A and in group B alone. The patients in group A showed significant improvement in pain score, TFS, and PROM of wrist flexion, extension, radial and ulnar deviation, as compare to group B.

Dellhag and colleagues conducted a clinical trial on 52 patients of rheumatoid arthritis and all were randomly placed into four groups, including exercise and wax bath, exercise only, wax bath only, and controls. All the patients were treated three times a week for four weeks. The group of patients treated with paraffin wax bath and followed by active exercises showed significant improvement as compared to the other groups treated with other techniques.^6^

Ayling and Marks carried out a systematic review on efficacy of paraffin wax bath for rheumatoid arthritic hand and critically examined whether paraffin wax is efficacious for this condition in light of this information. They found 4 randomized control trials, and 3 out of 4 reported that after 3-4 weeks of management, paraffin wax applications were accompanied by significant improvements in rheumatoid arthritic hand function when followed by exercise.^7^

Sandqvist and team conducted a clinical trial to determine the effect of paraffin wax bath combined with exercise, on one hand of 17 patients with scleroderma, while the other hand was treated with exercise only. They concluded that paraffin wax bath combined with exercise improved mobility, decrease stiffness, and increase elasticity.^8^

Valdes and Marik worked on a systematic review on the physical therapy management of osteoarthrtic hand, and they searched and evaluated evidence on multiple hand physical therapy interventions, including splinting, joint protection technique instruction, paraffin wax bath, exercises, and provision of a home exercise program. They concluded that literature supports the effectiveness of paraffin wax bath, joint protection instructions, and orthotic support for improvement in hand grip strength and function.^9^

Glasgow and team conducted a systematic review on mobilizing the stiff hand: combining theory and evidence to improve clinical outcomes. The purpose was to evaluate the available evidence on stiff hand. They concluded that mobilization exercise and splinting can prevent contractures in stiff hand.^10^

Sultana and colleagues carried out a systematic review on the role of mobilization after tendon transplant to evaluate the evidence on the role of mobilization after tendon transplant for the improvement of PROM pain at the wrist. They concluded on the basis of available studies of joint mobilization techniques, which are effective for pain management and improve function.^11^

## CONCLUSION

We conclude that paraffin wax bath combined with joint mobilization techniques are more effective in the physical therapy rehabilitation of post-traumatic stiff hand as compared to joint mobilization techniques alone.

## Notes

***Source of funding*** *:* Riphah International University Islamabad.
